# Inequity in cardiometabolic hospital admissions and blood screening in New Zealand Indigenous Māori with psychosis

**DOI:** 10.1192/bjo.2024.759

**Published:** 2024-09-24

**Authors:** Nathan J. Monk, Ruth Cunningham, James Stanley, Julie Fitzjohn, Melissa Kerdemelidis, Helen Lockett, Andre D. McLachlan, Richard J. Porter, Waikaremoana Waitoki, Cameron Lacey

**Affiliations:** Department of Māori/Indigenous Health Innovation, University of Otago, Christchurch, New Zealand; Department of Public Health, University of Otago, Wellington, New Zealand; Specialist Mental Health Service, Te Whatu Ora/Health New Zealand – Waitaha/Canterbury, New Zealand; Population Health Gain, Service Improvement and Innovation, Te Whatu Ora/Health New Zealand – Waitaha/Canterbury, New Zealand; Department of Public Health, University of Otago, Wellington, New Zealand; and Te Pou, Auckland, New Zealand; Centre for Health and Social Practice, Waikato Institute of Technology, Hamilton, New Zealand; Specialist Mental Health Service, Te Whatu Ora/Health New Zealand – Waitaha/Canterbury, New Zealand; and Department of Psychological Medicine, University of Otago, Christchurch, New Zealand; Faculty of Māori and Indigenous Studies, University of Waikato, Hamilton, New Zealand; Department of Psychological Medicine, University of Otago, Christchurch, New Zealand

**Keywords:** Psychosis, indigenous health, health equity, cardiometabolic health, blood screening

## Abstract

**Background:**

People with psychosis experience worse cardiometabolic health than the same-aged general population. In New Zealand, Indigenous Māori experiencing psychosis have greater risk of cardiometabolic and other physical health problems.

**Aims:**

To identify a cohort of adults accessing secondary mental health and addiction services in New Zealand, with a previous psychosis diagnosis as of 1 January 2018, and compare odds of hospital admission outcomes, mortality and receipt of cardiometabolic blood screening between Māori and non-Māori in the following 2 years.

**Method:**

Crude and adjusted logistic regression models compared odds of hospital admission outcomes, mortality and receipt of cardiometabolic blood screening (lipids and haemoglobin A_1c_) between Māori and non-Māori, occurring between 1 January 2018 and 31 December 2019.

**Results:**

A cohort (*N* = 21 214) of Māori (*n* = 7274) and non-Māori (*n* = 13 940) was identified. Māori had higher adjusted risk of mortality (odds ratio 1.26, 95% CI 1.03–1.54), and hospital admission with diabetes (odds ratio 1.64, 95% CI 1.43–1.87), cardiovascular disease (odds ratio 1.54, 95% CI 1.25–1.88) and any physical health condition (odds ratio 1.07, 95% CI 1.00–1.15) than non-Māori. Around a third of people did not receive recommended cardiometabolic blood screening, with no difference between Māori and non-Māori after covariate adjustment.

**Conclusions:**

Māori experiencing psychosis are more likely to die and be admitted to hospital with cardiovascular disease or diabetes than non-Māori. Because of the higher cardiometabolic risk borne by Māori, it is suggested that cardiometabolic screening shortfalls will lead to worsening physical health inequities for Māori experiencing psychosis.

People with psychosis experience premature mortality,^1^ caused by increased rates of physical illness, injury, poisoning and suicide.^2,3^ Increased physical illness among people with psychosis relates to a variety of complex risk factors, including cardiometabolic side-effects from antipsychotic medications.^4,5^ Higher rates of cardiovascular disease (CVD) and diabetes are of particular concern, especially because outcomes can be improved if these diseases are detected and treated early, and their poor prognoses if left unmanaged.^4,6^ In addition, people with psychosis can experience substantial barriers to accessing appropriate psychiatric and physical healthcare.^4,7,8^

In New Zealand, Indigenous Māori comprise approximately 17% of the population.^9^ Māori experience stark health inequity compared with non-Māori, and particularly when compared to New Zealand Europeans, who make up about 72% of the population.^10^ Māori health inequity arises within the context of historical and contemporary settler colonisation and racism, which has benefited primarily European settlers while over-exposing Māori to risk factors for poor health over multiple generations.^11^ Consequently, Māori live, on average, 7 years less than non-Māori.^12^

*Te Tiriti o Waitangi*/The Treaty of Waitangi (*Te Tiriti*), signed between Māori and the British Crown in 1840, is foundational to the uncodified constitution of New Zealand.^13^ Recommendations made by the Waitangi Tribunal are a contemporary basis for the implementation of *Te Tiriti* principles in health service delivery and policy.^14^ In particular, the principle of active protection guides ‘the Crown's responsibility to protect actively Māori health and wellbeing through the provision of health services … [and] requires the Crown to make available to Māori, as citizens, health services that reasonably and adequately attempt to close inequitable gaps in health outcomes with non-Māori’.^14^ The principle of partnership furthermore recognises the right of Māori to ‘choose how they organise themselves, and how or through what organisations they express their tino rangatiratanga [sovereignty]’.^14^ However, Māori-led health services have been strategically underfunded by the Crown,^14^ and Crown-run national health services have, to date, inadequately met Māori need.^15,16^

Recent New Zealand studies of people with psychosis and related disorders have reported that Māori have worse physical health and mortality outcomes than non-Māori. Among New Zealand adults with bipolar disorder, a recent study reported higher morbidity and mortality among Māori compared with non-Māori over a 5-year follow-up period.^17^ Similarly, inequities were found in cardiometabolic (diabetes and CVD-related) hospital admissions, as well as all-cause mortality, for *rangatahi* (young) Māori, emerging approximately 4–7 years following a first-episode psychosis diagnosis.^18^ However, no present data exist on Māori versus non-Māori physical health and mortality differences among all New Zealand adults experiencing psychosis (i.e. diagnosed with any psychotic disorder).

Recent New Zealand research on youth with psychosis has quantified relative socioeconomic privileges (in education, employment and criminal justice) among non-Māori youth when compared with *rangatahi* Māori in the year before being diagnosed with first-episode psychosis.^19^ To date, there is no evidence of physical health inequity for *rangatahi* Māori before or at first-episode psychosis diagnosis,^19^ but inequities emerge in the years immediately following first-episode psychosis, and continue to increase.^18^ *Rangatahi* Māori are also approximately twice as likely as non-Māori youth to be diagnosed with a psychotic disorder.^20^ Considering the inequitable physical health and premature mortality found for Māori in previous bipolar and psychosis studies,^17,18^ this is a critical health equity concern.

Recommendations for the physical health care of people with psychosis in New Zealand have been published,^21,22^ including current New Zealand Ministry of Health CVD risk assessment and management guidance,^23^ and implemented in practice guidelines used by clinicians, such as community health pathways for general practice and family medicine in New Zealand.^24^ Cardiometabolic blood screening is one important prevention tool for identifying risk of developing serious health conditions. To screen for CVD and diabetes risk, it is recommended that lipids and haemoglobin A_1c_ (HbA_1c_) are tested every 12 weeks during a person's first year taking antipsychotic medication, at least annually thereafter, and when responsibility for monitoring is being transferred from secondary care.^21,23^ Additionally, because of inequity in CVD risk burden for Māori, Pacific and South Asian peoples in the general population, it is recommended that CVD screening in the general population starts 15 years earlier than for people without known risk factors.^23^ However, although national population CVD screening guidelines include serious mental illness (e.g. schizophrenia) as a significant risk factor that warrants CVD screening, New Zealand has no formal national guidelines or programme for CVD or metabolic screening for people with psychosis. Conversely, the United Kingdom, for instance, has a free annual physical health screening programme for people with psychosis.^25^

Because of heightened cardiometabolic risk, all people experiencing psychosis should receive routine cardiometabolic blood screening. However, limited resourcing and barriers to care mean that it is likely there are gaps in screening coverage.^7,8^ Because of physical health inequity, Māori are a priority group for cardiometabolic health screening and intervention in the general population.^23^ Among people experiencing psychosis, Māori appear to experience similar physical health inequities to those observed for Māori in the general population.^17,18^ Thus, although cardiometabolic screening is recommended for all people experiencing psychosis, the likely inequitable cardiometabolic risk borne by Māori experiencing psychosis renders this group high priority.

However, there are reasons to expect inequitable cardiometabolic screening coverage for Māori. A Māori-led body of qualitative research has voiced experiences of structural racism in the health system as a strong barrier to receiving general care.^16^ *Rangatahi* Māori with early-course psychosis have reported both racism and psychosis-related prejudice when engaging with health services.^26^ Other recent work has documented structural barriers which prevent Māori with bipolar disorder from receiving appropriate healthcare in New Zealand.^15,27,28^ These barriers for Māori sit in addition to established barriers to physical health care for all people with psychosis.^7,8^

Additionally, building on recent research on physical health equity and mortality among adults experiencing bipolar disorder^17^ and youth following first-episode psychosis,^18^ physical health and mortality equity data are needed for adults experiencing psychosis.

## Aims

This study uses linked national health data for people aged 16–64 years, who are in contact with secondary mental health and addiction services, with a psychosis diagnosis. It builds on previous research by assessing both physical health and mortality measures and receipt of routine cardiometabolic blood screening (lipids and HbA_1c_) as a preventative physical healthcare measure. Over a 2-year study period (1 January 2018 to 31 December 2019), the aims of this study are:

- Compare Māori and non-Māori risk of hospital admission (including CVD- and diabetes-specific hospital admissions) and mortality.
- Compare Māori and non-Māori odds of receiving cardiometabolic blood screening (lipids and HbA_1c_).
- Compare the above outcomes between Māori dispensed antipsychotic medication and Māori not dispensed antipsychotic medication.

## Method

### Ethics

The authors assert that all procedures contributing to this work comply with the ethical standards of the relevant national and institutional committees on human experimentation and with the Helsinki Declaration of 1975, as revised in 2008. All procedures involving human patients were approved by University of Otago Ethics Committee (reference number HD22/089).

### Participants

The study cohort was formed based on data from the Programme for the Integration of Mental Health Data (PRIMHD) as part of Te Whatu Ora/Health New Zealand National Collections data. PRIMHD contains data from all publicly funded specialist mental health and addiction service contact in New Zealand since July 2007.^29^ From PRIMHD, we identified a cohort of people (Māori and non-Māori) aged 16–64 years, with a recorded psychosis diagnosis on or before 31 December 2017, and who were alive on 1 January 2018. Cohort inclusion was capped at a maximum age of 64 years as mental health and addiction service use data are incomplete for people aged 65 years and older.^30^ Inclusion codes were DSM-IV and ICD-10 diagnostic codes for schizophrenia, type 1 bipolar disorder, schizoaffective disorder, depressive disorder with psychotic symptoms, organic psychotic disorder, substance-induced psychotic disorder and psychosis not otherwise specified. People with psychosis were identified via both DSM-IV and ICD-9/10 regularly across the cohort identification period. Different regions transitioned from using ICD-9 to ICD-10 at different times during the time period. Diagnostic codes are given in the Supplementary material (Supplementary Table 1 available at https://doi.org/10.1192/bjo.2024.759). No differentiation was made between in-patient and out-patient events. Only primary and other relevant diagnoses were used for study inclusion (provisional diagnoses were excluded). People were excluded if they had no recorded contact with mental health and addiction services during the 2-year study period (1 January 2018 to 31 December 2019); this was done so the cohort includes only people for whom there was demonstrated opportunity to perform cardiometabolic screening (i.e. contact with a clinician). Participant consent is not required to analyse de-identified National Collections records.^31^

### Measures

Data were linked between National Collections data-sets^32^ via an encrypted National Health Index (NHI) identifier, covering hospital admission (National Minimum Dataset (NMDS)), mortality (Mortality Collection), laboratory (Laboratory Claims Collection), mental health and addiction service use (PRIMHD) and pharmaceutical dispensing (Pharmaceutical Collection). Data were extracted from 1 January 2018 to 31 December 2019.

In New Zealand, in-patient admission diagnostic coding is performed by trained coders who code each diagnosis based on a standardised set of rules applied to the clinical file. In out-patient specialist care, a diagnosis must be entered by clinicians either at discharge or after 3 months of care.

Ethnicity and other demographic data were obtained from the master NHI data-set. Ethnicity was collected during healthcare interactions, with protocols for collection stipulating that this should be self-identified ethnicity.^33^ Māori patients were identified by prioritised ethnicity; patients were categorised as Māori if they were recorded as Māori, regardless of any other recorded ethnicities. All other patients were categorised as non-Māori. To align with *Te Tiriti*,^14^ all main analyses compared Māori and non-Māori.

#### Hospital admissions

Hospital admissions during the study period were defined with diagnostic codes recorded in the NMDS. The NMDS contains all public and some private hospital admission data. Most hospital care, in particular all acute admissions, takes place in the public system, so this gives good coverage of hospital admission events. All hospital admissions were filtered to only include medical and surgical admissions (Health Specialty Codes beginning ‘M’ or ‘S’), thus excluding psychiatric admissions. Four categories of admission were defined based on presence of specific ICD codes as primary or additional diagnostic codes: (a) physical health, (b) injury/poisoning, (c) CVD and (d) diabetes.

##### Physical health hospital admissions

Physical health covered a broad category of admissions with any recorded physical health condition diagnosis, excluding conditions related to pregnancy and childbirth (ICD-9 codes 001–289, 320–629, 680–739; ICD-10 codes A00–E89, G00–N99).

##### Injury/poisoning hospital admissions

These were defined as admissions with any recorded diagnosis of an injury (including burns) or poisoning (ICD-9 codes 800–999; ICD-10 codes S00–T88).

##### CVD-related hospital admissions

These were defined as admissions where any relevant diagnosis of CVD was recorded (see Supplementary Table 2 for codes).

##### Diabetes hospital admissions

These were defined as admissions where any relevant diagnosis of diabetes (ICD-9 code 250; ICD-10 codes E10–E11; gestational diabetes was excluded) was recorded.

#### Cardiometabolic blood screening

The Laboratory Claims collection contains claim and payment information for community laboratory tests that have been processed by the HealthPAC General Transaction Processing System, and laboratory test information from Pegasus Health and Medlab South.^34^ From the data, records of lipids and HbA_1c_ tests were identified. Lipids screening was defined as completed if any relevant test was recorded (i.e. any of triglycerides, total cholesterol, high-density lipoprotein cholesterol, low-density lipoprotein cholesterol or total cholesterol/high-density lipoprotein ratio).

#### Mental healthcare

Several aspects of mental health and addiction care during the study period were identified from PRIMHD and the Pharmaceutical Collection data: (a) placed under Mental Health (Compulsory Assessment and Treatment) Act 1992 (any section); (b) psychiatric in-patient admission (intensive care, acute or subacute); (c) use of seclusion (within an in-patient setting) and (d) dispensing of typical and/or atypical antipsychotic medications.

#### Physical multimorbidity at study entry (M3)

Physical multimorbidity at study entry (1 January 2018) was assessed with a modified version of the M3 multimorbidity index.^35^ Multimorbidity is quantified with ICD-10 diagnoses recorded in hospital admission data over a 5-year lookback period from study entry. The M3 assigns weights from 55 medical conditions based on their predictiveness of mortality. The present study concerns physical health status, so five psychiatric categories were excluded from the M3 score calculation (major psychiatric disorder, mental and behavioural disorder, anxiety and behavioural disorder, drug and alcohol codes). To describe physical health at study entry, M3 scores were categorised into 0, >0 to <1, 1 to <2, and ≥2. A score of zero indicates that no relevant diagnostic codes were present in hospital admission data; higher scores indicate presence of condition(s) associated with higher mortality.

#### Covariates

Gender and date of birth were obtained from the NHI data-set. Socioeconomic deprivation was assessed with New Zealand Index of Deprivation 2018 quintiles, which indicates the relative deprivation for geographic areas of New Zealand, based on the 2018 New Zealandcensus.^36^ Regression analyses were adjusted for potential confounding by gender, age and socioeconomic deprivation. The New Zealand Index of Deprivation 2018 quintiles were treated as ordinal in regression models. Age was treated as linear and scaled into 5-year increments to aid interpretability of odds ratios. Both crude and adjusted estimates are presented because, for screening in particular, indicated ages differ between Māori and non-Māori (in the general population), so adjustment for age may obscure important differences in indicated screening.

Non-psychotic psychiatric comorbidities were identified from the NMDS and PRIMHD. Psychiatric comorbidities were categorised into depression, anxiety, substance disorders and personality disorders (see Supplementary Table 3 for codes).

### Analysis

All data cleaning and analysis was performed in the R software environment for Windows (R 4.1, R Institute, Vienna, Austria; see https://cran.r-project.org/bin/windows/base/). Reporting follows the Strengthening the Reporting of Observational Studies in Epidemiology (STROBE) guidelines.^37^

#### Descriptive analyses

Descriptive analyses of Māori and non-Māori cover demographic, clinical and service use characteristics, including multimorbidity and hospital admissions before study entry. These descriptive analyses are presented as profiles (counts and percentages): no formal statistical comparisons are made, as the objective of this table is to describe general differences by ethnicity that may be relevant for considering confounding.^37^

#### Māori versus non-Māori comparisons

Logistic regression models were estimated to compare Māori and non-Māori in risk of hospital admission, mortality and cardiometabolic blood screening over the 2-year study period. Because of the cardiometabolic risks associated with antipsychotic medication, further logistic regression models restricted to Māori were used to compare cardiometabolic admissions and blood screening in Māori receiving antipsychotic medication with Māori not receiving antipsychotic medication. Further logistic regression models also compared Māori males and Māori females in risk of hospital admission, mortality and blood screening. Logistic regression models assume follow-up time is consistent across the sample.

#### Supplementary analysis

A supplementary analysis was performed to quantify likely inequities for Pacific peoples, who sit outside the scope of the present research aims, but who also experience significant health inequities in New Zealand. Although these findings are not directly related to the aims of the present study, the study data-set presented an opportunity to disseminate supplementary findings in this underresearched area. Crude and adjusted analysis of hospital admission, mortality and blood screening outcomes were re-estimated for three ethnic groups: Māori, Pacific peoples and New Zealand European. Pacific peoples (*n* = 1769) and New Zealand European (*n* = 9702) ethnicities were identified by using the NHI data-set.^33^

## Results

**Fig. 1:**
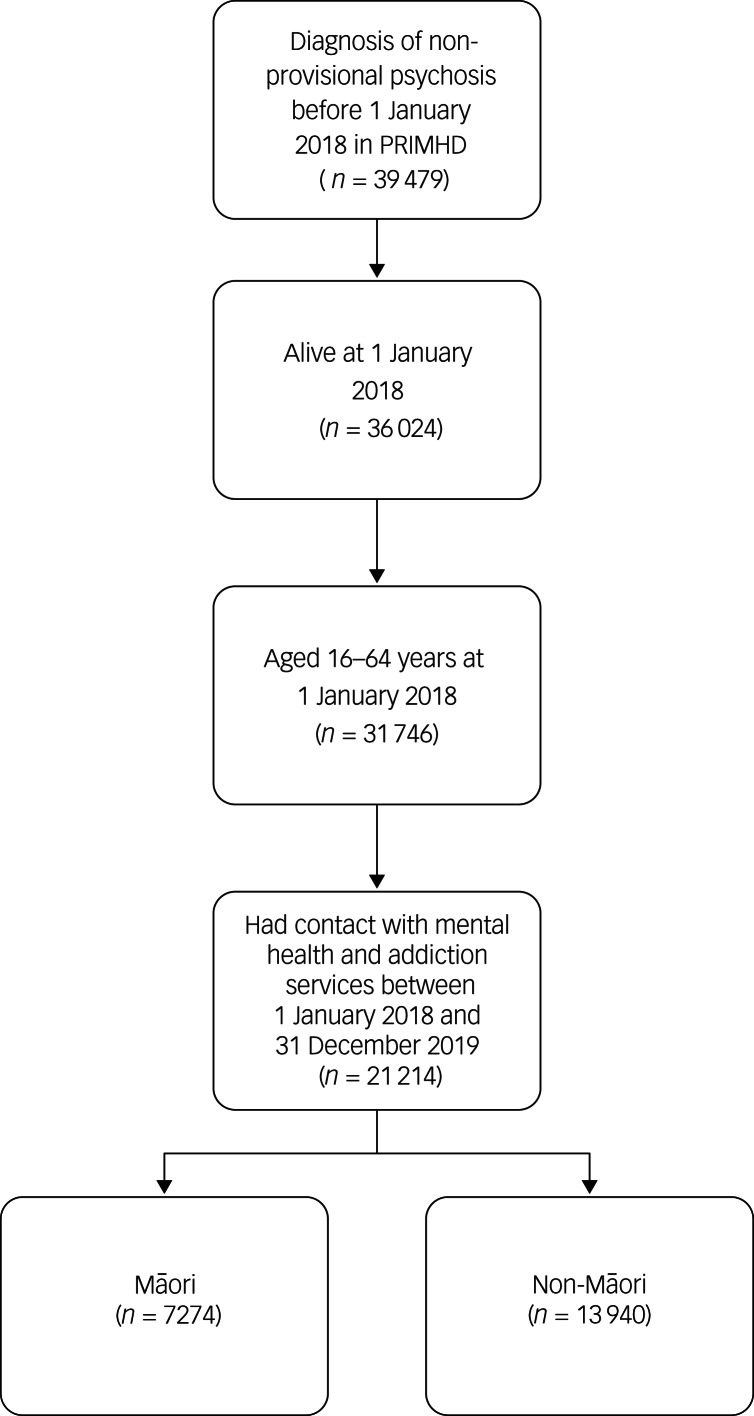
Cohort identification steps. PRIMHD, Programme for the Integration of Mental Health Data.

A total of 21 214 people were included in the study cohort, according to the inclusion criteria (see Fig. 1). About a third of the study cohort were Māori (*n* = 7274, 34.3%).

Table 1 describes the demographic and clinical characteristics of Māori and non-Māori at study entry. Relative to non-Māori, Māori tended to be younger, exposed to more socioeconomic deprivation and have higher physical multimorbidity at study entry. Māori were more likely to have diagnoses of schizophrenia, substance-induced psychosis, schizoaffective disorder and unspecified psychosis; and less likely to have diagnoses of bipolar disorder and depression with psychosis. Māori were also more likely to have diagnoses of substance use and personality disorders, and less likely to have diagnoses of major depression and anxiety disorder. Māori were more likely to be dispensed antipsychotic medication (typical and atypical) and be placed under the Mental Health Act, and were nearly twice as likely to experience seclusion.

**Table 1:** Demographic and clinical characteristics of Māori and non-Māori at 1 January 2018

	Māori		Non-Māori	
	*n*	%	*n*	%
Total	7274		13 940	
Gender^a^				
Male	4486	61.7	8028	57.6
Female	2786	38.3	5904	42.4
Mean (s.d.) age, years	38.1	(11.9)	41.9	(12.5)
Age group, years				
16–29	2085	28.7	2876	20.6
30–44	2854	39.2	4791	34.4
45–64	2335	32.1	6273	45.0
Deprivation quintile				
1	280	3.8	1442	10.3
2	570	7.8	2064	14.8
3	911	12.5	2667	19.1
4	2016	27.7	4029	28.9
5	3476	47.8	3716	26.7
Prior psychosis diagnoses^b^				
Schizophrenia	5289	72.7	8097	58.1
Bipolar disorder type 1	2212	30.4	5252	37.7
Depression with psychosis	581	8.0	1579	11.3
Substance-induced psychosis	1727	23.7	1626	11.7
Schizoaffective	2149	29.5	3317	23.8
Other	1728	23.8	3170	22.7
Organic	107	1.5	192	1.4
Not otherwise specified psychosis	3322	45.7	5431	39.0
Not otherwise specified psychosis only	293	4.0	623	4.5
Other prior psychiatric diagnoses				
Substance use disorders	5000	68.7	5937	42.6
Depression	1309	18.0	3368	24.2
Anxiety	841	11.6	2885	20.7
Personality disorders	1795	24.7	2861	20.5
Physical multimorbidity^c^				
M3 mean (s.d.)	0.22	(0.48)	0.19	(0.45)
0	4989	68.6	9980	71.6
>0 to <1	1765	24.3	3120	22.4
1 to <2	398	5.5	638	4.6
≥2	120	1.6	200	1.4
Prior cardiometabolic hospital admission				
Cardiovascular disease	179	2.5	393	2.8
Diabetes	734	10.1	941	6.8
Mental health treatment during study period				
Treated under the Mental Health Act	2132	29.3	3124	22.4
Seclusion	503	6.9	524	3.8
Typical antipsychotic medication	1158	15.9	1404	10.1
Atypical antipsychotic medication	3410	46.9	5636	40.4
Any antipsychotic medication	4202	57.8	6557	48.6

a. A total of ten people (two Māori and eight non-Māori) were recorded as other or unknown gender.

b. All psychiatric diagnoses use an unrestricted lookback period.

c. M3 score and prior hospital admissions for cardiovascular disease and diabetes used a 5-year lookback period from study entry.

Risk of hospital admission, mortality and cardiometabolic blood screening during the study period are presented in Table 2. Approximately a quarter of the cohort were admitted to hospital with a physical health condition over the study period, and approximately a fifth were admitted to hospital with an injury or poisoning. Cardiometabolic admissions were uncommon, but more common among Māori that non-Māori. After adjustment for confounding by age, gender and socioeconomic deprivation, Māori were more likely to be admitted to hospital with any physical health condition (odds ratio 1.07, 95% CI 1.00–1.15), CVD (odds ratio 1.54, 95% CI 1.25–1.88) and diabetes (odds ratio 1.64, 95% CI 1.43–1.87), and had a higher risk of mortality (odds ratio 1.26, 95% CI 1.03–1.54).

**Table 2:** Risk of hospital admission, mortality and blood screening events for Māori (*n* = 7274) compared with non-Māori (*n* = 13 940), during the 2018–2019 study period

	Māori, *n* (%)	Non-Māori, *n* (%)	Crude odds ratio (95% CI)	Adjusted odds ratio^a^ (95% CI)
Physical health admission	1786 (24.6)	3544 (25.4)	0.95 (0.89–1.02)	1.07 (1.00–1.15)
Injury/poisoning admission	1570 (21.6)	3148 (22.6)	0.94 (0.88–1.01)	1.02 (0.95–1.09)
Cardiovascular admission	168 (2.3)	281 (2.0)	1.15 (0.95–1.39)	1.54 (1.25–1.88)
Diabetes admission	427 (5.9)	617 (4.4)	1.35 (1.19–1.53)	1.64 (1.43–1.87)
Mortality	163 (2.2)	299 (2.1)	1.05 (0.86–1.27)	1.26 (1.03–1.54)
Lipids screening	4373 (60.1)	8718 (62.5)	0.90 (0.85–0.96)	1.02 (0.96–1.09)
Haemoglobin A_1c_ screening	4770 (65.6)	9629 (69.1)	0.85 (0.80–0.91)	0.97 (0.91–1.04)

a. Adjusted for age, gender and socioeconomic deprivation.

Of the 4718 people admitted to hospital with injury or poisoning, 825 were coded as self-harm: 236 Māori (3.2% of total Māori) and 589 non-Māori (4.2% of total non-Māori). Cause of death data were only available for the first year of follow-up, as 2019 cause of death data were not yet available at the time of extraction. Of the 214 deaths in 2018, 125 (58.4%) were attributed to a medical cause: 35 to cancer, 35 to CVD, 16 to respiratory conditions, 13 to metabolic/nutritional/endocrine conditions (e.g. diabetes), and 26 to other physical health conditions. The remaining 89 deaths were from non-medical events: 47 from suicide, 28 from accidental poisoning (18 from medications; ICD-10 code X41) and 14 from other events (e.g. transport accident). During the study period, there were 32 895 total hospital admissions involving 10 322 unique patients; 10 386 admissions were same-day discharges.

Less than two-thirds of Māori received recommended screening for lipids (60.1%) and HbA_1c_ (65.6%). Māori were less likely receive lipids (crude odds ratio 0.90, 95% CI 0.85–0.96) and HbA_1c_ (crude odds ratio 0.85, 95% CI 0.80–0.91) blood screening than non-Māori. However, after adjustment for differences in age, gender and socioeconomic deprivation, there was no statistical difference between Māori and non-Māori in rates of screening for lipids (odds ratio 1.02, 95% CI 0.96–1.09) or HbA_1c_ (odds ratio 0.97, 95% CI 0.91–1.04). In adjusted models, higher socioeconomic deprivation (odds ratio 1.05, 95% CI 1.02–1.07) and older age (odds ratio 1.23, 95% CI 1.22–1.25; scaled to represent a 5-year age difference) were significant positive predictors of lipids screening receipt. Older age (odds ratio 1.20, 95% CI 1.18–1.21) and female gender (odds ratio 1.44, 95% CI 1.35–1.53) were significant positive predictors of HbA_1c_ screening receipt.

Table 3 compares receipt of blood screening and diabetes- and CVD-related hospital admissions between Māori dispensed antipsychotic medication and Māori not dispensed antipsychotic medication. Māori who were dispensed antipsychotic medication (mean 38.5 years, s.d. = 11.6) tended to be older than Māori not dispensed antipsychotic medication (mean 37.5 years, s.d. = 12.2; *t*[6417.7] = 3.45, *P* < 0.001). Māori males were also more likely to be dispensed antipsychotic medication than Māori females (*χ*^2^[1, *n* = 7272] = 64.9, *P* < 0.001). After covariate adjustment, Māori who were dispensed antipsychotic medication were more likely to have screening completed for lipids (odds ratio 1.51, 95% CI 1.37–1.67) and HbA_1c_ (odds ratio 1.37, 95% CI 1.24–1.52), more likely to be admitted to hospital with diabetes (odds ratio 1.60, 95% CI 1.29–1.98) and less likely to be admitted to hospital with CVD (odds ratio 0.72, 95% CI 0.53–0.99).

**Table 3:** Risk of cardiometabolic hospital admission and blood screening for Māori, compared between those dispensed (*n* = 4202) and not dispensed (*n* = 3072) antipsychotic medication during the 2018–2019 study period

	Māori dispensed antipsychotics, *n* (%)	Māori not dispensed antipsychotics, *n* (%)	Crude odds ratio (95% CI)	Adjusted odds ratio (95% CI)^a^
Cardiovascular admission	85 (2.0)	83 (2.7)	0.74 (0.55–1.01)	0.72 (0.53–0.99)
Diabetes admission	288 (6.9)	139 (4.5)	1.55 (1.26–1.92)	1.60 (1.29–1.98)
Lipids screening	2713 (64.6)	1660 (54.0)	1.55 (1.41–1.70)	1.51 (1.37–1.67)
Haemoglobin A_1C_ screening	2882 (68.6)	1888 (61.5)	1.37 (1.24–1.51)	1.37 (1.24–1.52)

a. Adjusted for age, gender and socioeconomic deprivation.

Further analysis compared outcomes for Māori males and Māori females within the study cohort. These comparisons were adjusted for age, as Māori females (mean 39.7 years, s.d. = 12.3) were older than Māori males (mean 37.1 years, s.d. = 11.5; *t*[5616.5] = 9.22, *P* < 0.001). Māori females were more likely to be admitted to hospital with any physical health condition (odds ratio 1.66, 95% CI 1.48–1.85), injury/poisoning (odds ratio 1.55, 95% CI 1.38–1.74) and diabetes (odds ratio 1.71, 95% CI 1.40–2.09). No difference was found for CVD-related hospital admissions (odds ratio 0.98, 95% CI 0.71–1.34). Māori females appeared less likely to die, although the estimate for this difference was imprecise and did not exclude the null (odds ratio 0.77, 95% CI 0.55–1.07). Māori females were less likely to receive lipids screening (odds ratio 0.89, 95% CI 0.80–0.98), but more likely to receive HbA_1c_ screening (odds ratio 1.26, 95% CI 1.14–1.40).

Supplementary analysis (see Supplementary Table 4) showed Pacific peoples with psychosis diagnoses having higher adjusted risk of hospital admission with diabetes (odds ratio 2.79, 95% CI 2.24–3.46) and CVD (odds ratio 1.86, 95% CI 1.31–2.60) than New Zealand European people. Pacific peoples had significantly higher crude and adjusted estimates for lipids (adjusted odds ratio 2.04, 95% CI 1.81–2.30) and HbA_1c_ (adjusted odds ratio 1.75, 95% CI 1.55–1.97) screening receipt compared with New Zealand European people with psychosis.

## Discussion

### Summary and interpretation of results

In this large cohort of 21 214 adults with psychosis who access secondary mental health and addiction services in New Zealand, Māori had higher adjusted risk of mortality, as well as higher risk of hospital admission with CVD, diabetes and any physical health condition than non-Māori. Greater inequities were found for cardiometabolic (CVD- and diabetes-related) admissions than for the broader physical health admissions category. Māori also had higher physical multimorbidity (M3 scores) at the beginning of the study period, suggesting an overall poorer state of health. Māori were less likely to receive cardiometabolic blood screening (lipids and HbA_1c_), although these differences became null after adjusting for gender, age and socioeconomic deprivation.

Overall, approximately a third of people did not receive any recommended cardiometabolic blood screening during the 2-year study period. It should be noted that only people with recorded mental health and addiction service contact were included in the analysis. Thus, this gap in blood screening coverage is not accounted for by people falling out of service contact; this gap represents people who had contact with specialist services, and for whom screening was likely possible.

The estimated cardiometabolic blood screening rates are broadly comparable to uptake on annual physical health checks (which include cardiometabolic blood screening) for people experiencing psychosis in the UK. During the most recent recorded year, 58.5% of eligible people received their annual physical health check in the UK.^38^ Unlike New Zealand, the UK provides free annual physical health checks for people aged 18 years and over experiencing psychosis.^25^ However, despite being free in the UK, uptake of physical health checks appears broadly in line with cardiometabolic blood screening coverage among patients in New Zealand. So, although cost is a significant barrier for many people accessing healthcare,^39^ UK data suggest that removing cost alone will not be an adequate solution to achieving universal screening coverage.

As a human rights and quality-of-care issue, reduction of physical healthcare barriers for people experiencing psychosis should be considered urgent. Health policy makers and practitioners may look to, for example, the Annual Diabetes Review as a formal template for recommended routine health screening.^40^ Although annual monitoring is recommended for people with psychosis in the general New Zealand CVD risk assessment guidelines^23^ and academic literature,^21^ no physical health monitoring guidelines or programmes for people with psychosis have been formalised in New Zealand. The wider research project, which comprises the present study, will produce recommendations on how physical health screening can be improved for people with psychosis in New Zealand, particularly Māori. It is clear from the present results that contact with mental health and addiction services alone does not ensure completion of recommended cardiometabolic blood screening, so more work in this area is needed.

Considering the increased physical health risks for Māori with psychosis, screening for cardiometabolic conditions is a priority. However, we found that only 60.1% of Māori received recommended lipids screening, and only 65.6% of Māori received recommended HbA_1c_ screening during the study period. Among Māori, those receiving antipsychotic medication were more likely to have lipids (64.6%) and HbA_1c_ (68.6%) screening completed. However, approximately a third of Māori receiving antipsychotics still did not have any blood screening completed over the 2-year study period. Māori and non-Māori had comparable adjusted odds of having blood screening completed. Age was a significant predictor of both lipids and HbA_1c_ screening, so the crude finding that Māori are less likely to receive recommended blood screening is explained, in part, by younger age (Māori were nearly 4 years younger, on average, in this cohort). Against the background of enhanced risk for related health outcomes for Māori, as well as apparently higher rates of antipsychotic dispensing (57.8% compared with 48.6% for non-Māori in this cohort), this is a healthcare inequity. Facilitating higher screening, particularly soon after psychosis diagnosis,^18^ should be a target to improve the physical health of Māori with psychosis. Otherwise, it appears likely that the present shortfall in cardiometabolic screening will disproportionately harm Māori.

Improving the physical health of Māori with psychosis is mandated by the principles of *Te Tiriti*.^14^ We suggest this should begin with intentionally facilitating culturally safe healthcare. Recently, Māori with psychosis have reported both structural and interpersonal racism in the New Zealand health system,^15,26–28^ coupled with diagnostic overshadowing.^8^ Accessible physical healthcare for Māori with psychosis involves clear pathways through care, improving the clinical culture (as expressed by staff) and the enhancement of support networks alongside people experiencing psychosis.^28^ *Kaupapa* Māori healthcare models, such as the *Meihana* model and *Hui* process,^41,42^ enshrine these facilitators in formal service delivery frameworks based in *tikanga* (customs) and *te ao* (worldview) Māori. Enhancement of Māori with psychosis will require the privileging of *Kaupapa* Māori healthcare providers, which have been historically underfunded by the Crown.^14^ To attempt to address these issues, *Te Aka Whaiora*/Māori Health Authority, was set up as an agency specifically responsible for health services for Māori. However, a new New Zealand Government started work to disestablish this entity in late 2023.^43^

Among Māori, we found that antipsychotic medication was associated with increased risk for diabetes hospital admission and reduced risk for CVD hospital admission. The lower rate of CVD hospital admission is surprising, given the well-established cardiometabolic risks of antipsychotic medications.^4,5,21^ However, one recent within-person analysis reported no difference in hospital admission risk for individuals during antipsychotic exposure periods compared to non-exposure periods, and also reported a reduction in cardiovascular mortality during antipsychotic exposure periods.^44^ Another recent study reported that antipsychotic use is associated with greater adherence to cardiometabolic medications,^45^ suggesting a complex interplay of pharmacology and behaviours driving the observed patterns.

The overall effect of antipsychotic medications on physical morbidity and mortality appears complex. Despite their cardiometabolic side-effects, antipsychotics reduce all-cause mortality in people with schizophrenia.^46^ However, people with psychosis who take antipsychotic medications have much worse physical health than the same-age general population.^4^ Consequently, in addition to routine clinical monitoring (e.g. blood screening) and intervention, integrated care approaches appear a promising mode of support for people experiencing psychosis, and their support networks, to improve physical health. These approaches take a wider scope to improving the overall health of people with psychosis, typically involving support from a team of multidisciplinary specialists.^47,48^ Early pilot evaluations of multidisciplinary support programmes have shown promise in New Zealand, including for Māori.^47^ However, further rollout and evaluation is needed.

Supplementary analyses (Supplementary Table 4) identified that Pacific peoples experiencing psychosis receive cardiometabolic blood screening at substantially higher rates than both Māori and New Zealand European people experiencing psychosis. These findings demonstrate that it is possible to reach a higher-risk ethnic group with equitable screening coverage. It should be considered what factors are facilitating better coverage for Pacific peoples, and how these may transfer to addressing the present concern of improving Māori blood screening. Based on the reported experiences on Māori with psychosis in the health system described above,^15,26,27^ we suggest that this begins with honouring *Te Tiriti* principles of partnership, equity, active protection and options^14^ in healthcare through funding *Kaupapa* Māori health services and maintaining educational and professional pathways for Māori in the health system.

### Strengths and limitations

The study used a national cross-section of people experiencing psychosis and in contact with publicly funded mental health and addiction services between 1 January 2018 and 31 December 2019. In New Zealand, nearly all psychiatric care related to psychotic mental disorders occurs in the public health system, at least at some point during care, so the vast majority of eligible patients will have been included in this study. Encrypted identifiers enabled linkage of patient records across multiple National Collections data-sets, providing good national coverage of both people and health/healthcare events. Notably, the present cohort contains only active adults accessing secondary mental health and addiction services in New Zealand, and thus screening gaps in this group are modifiable through clinicians in these settings. However, a group of 10 532 people were excluded from the study, as they did not have contact with mental health and addiction services during the study period. This exclusion was made because it was not possible to determine whether this group had no contact with specialist services because they had no need (e.g. they may have had very mild and possibly brief psychosis, and therefore may not be indicated for blood screening) or because they had, for example, severe negative symptoms. Without specialist service contact, this group will be harder to reach; however, we cannot say what their health status or need may be.

There are some limitations to this study. First, cardiometabolic blood screening was chosen for this analysis, because of the high-quality and accessible national data. However, there are other important screening tools and aspects of physical healthcare that were not able to be accounted for because there was no or less accessible national-level data (e.g. blood pressure, weight/body mass index and full CVD risk assessment). Although such work will be more challenging with present data constraints, future research drawing on different data-sets is needed to document the care Māori with psychosis are receiving over time, across healthcare settings (i.e. in primary care and across various mental health services). Second, to best align with *Te Tiriti*, Māori were referenced against all non-Māori in main analyses. However, health status and healthcare received will vary between non-Māori ethnic groups (see e.g. Supplementary Table 4). Third, although we adjusted for social deprivation, we were unable to adjust for more granular predictors of poor health (e.g. tobacco use). Finally, the NHI ethnicity measure used in this study has been reported to undercount Māori by 16%,^49^ meaning some Māori are likely incorrectly classified as non-Māori. This may lead to underestimates of differences by ethnicity.

In conclusion, indigenous Māori adults (aged 18–64 years) with psychosis who access secondary mental health and addiction services in New Zealand had greater adjusted risk of being admitted to hospital with diabetes or CVD than non-Māori during a 2-year study period (1 January 2018 to 31 December 2019). Māori and non-Māori received recommended cardiometabolic blood screening at similar rates after adjusting for covariates. Overall failure to complete cardiometabolic blood screening for at least three out of every ten people with psychosis in New Zealand will lead to substantial cardiometabolic risk going undetected. Because of the increased physical health burden borne by Māori with psychosis, this failure is likely to perpetuate and worsen cardiometabolic health and mortality inequities for Māori experiencing psychosis. The New Zealand health system must seek to address these gaps in cardiometabolic screening for people with psychosis, so practitioners can intervene on modifiable causes of physical illness. Facilitating culturally safe physical health services for Māori with psychosis is one area that requires deliberate attention.

The title of this wider research project, *Te Pu Korokoro*, is derived from a *pūrākau*. *Pūrākau* are a form of narrative or story that provide context and connections between the present and the past. They are noted to contain philosophical thought, epistemological constructs, cultural codes and worldviews that are vital for collective identity as Māori.^50^ Many *pūrākau* portray the characteristics of different animals or other elements in the *taiao* (environment) that can benefit the well-being and prosperity of people. The *pūrākau* of *Te Pu Korokoro* relates the experience of Māori experiencing psychosis to that of *kuaka* (godwit) falling from the safety of an air pocket into harsh winds during migration. The present paper quantifies some physical health- and care-related aspects of this experience. Like the migrating *kuaka*, Māori with psychosis must be supported on their own journey. Ultimately, the aim of *Te Pu Korokoro* is to deliver recommendations on how the New Zealand health system can best support these journeys.

## Supporting information

Monk et al. supplementary material Monk et al. supplementary material

## Data Availability

The data are not publicly available due to ethical compliance regarding de-identified national health service records.

## References

[1] Walker ER, McGee RE, Druss BG. Mortality in mental disorders and global disease burden implications: a systematic review and meta-analysis. JAMA Psychiatry 2015; 72(4): 334–41. 25671328 10.1001/jamapsychiatry.2014.2502 PMC4461039

[2] Yuen K, Harrigan SM, Mackinnon AJ, Harris MG, Yuen HP, Henry LP, et al. Long-term follow-up of all-cause and unnatural death in young people with first-episode psychosis. Schizophr Res 2014; 159(1): 70–5. 25151199 10.1016/j.schres.2014.07.042

[3] Simon GE, Stewart C, Yarborough BJ, Lynch F, Coleman KJ, Beck A, et al. Mortality rates after the first diagnosis of psychotic disorder in adolescents and young adults. JAMA Psychiatry 2018; 75(3): 254–60. 29387876 10.1001/jamapsychiatry.2017.4437 PMC5885951

[4] Firth J, Siddiqi N, Koyanagi A, Siskind D, Rosenbaum S, Galletly C, et al. The Lancet Psychiatry Commission: a blueprint for protecting physical health in people with mental illness. Lancet Psychiatry 2019; 6(8): 675–712. 31324560 10.1016/S2215-0366(19)30132-4

[5] Galletly C, Foley DL, Waterreus A, Watts GF, Castle DJ, McGrath JJ, et al. Cardiometabolic risk factors in people with psychotic disorders: the second Australian national survey of psychosis. Aust N Z J Psychiatry 2012; 46(8): 753–61. 22761397 10.1177/0004867412453089

[6] Correll CU, Solmi M, Veronese N, Bortolato B, Rosson S, Santonastaso P, et al. Prevalence, incidence and mortality from cardiovascular disease in patients with pooled and specific severe mental illness: a large-scale meta-analysis of 3,211,768 patients and 113,383,368 controls. World Psychiatry 2017; 16(2): 163–80. 28498599 10.1002/wps.20420 PMC5428179

[7] Cunningham R, Imlach F, Haitana T, Every-Palmer S, Lacey C, Lockett H, et al. It's not in my head: a qualitative analysis of experiences of discrimination in people with mental health and substance use conditions seeking physical healthcare. Front Psychiatry 2023; 14: 1285431. 37908598 10.3389/fpsyt.2023.1285431 PMC10613695

[8] Cunningham R, Imlach F, Lockett H, Lacey C, Haitana T, Every-Palmer S, et al. Do patients with mental health and substance use conditions experience discrimination and diagnostic overshadowing in primary care in Aotearoa New Zealand? Results from a national online survey. J Prim Health Care 2023; 15(2): 112–21. 37390032 10.1071/HC23015

[9] Statistics New Zealand. *Māori Population Estimates: At 30 June 2023*. New Zealand Government, 2023 (https://www.stats.govt.nz/information-releases/maori-population-estimates-at-30-june-2023/#:~:text=At%2030%20June%202023%3A,percent%20of%20the%20national%20populations).

[10] Ministry of Health. Wai 2575 Māori Health Trends Report. Ministry of Health, 2019 (https://www.health.govt.nz/publications/wai-2575-maori-health-trends-report).

[11] Reid P, Cormack D, Paine SJ. Colonial histories, racism and health – the experience of Māori and Indigenous peoples. Public Health 2019; 172: 119–24. 31171363 10.1016/j.puhe.2019.03.027

[12] Ministry of Health. Tatau Kahukura: Māori Health Statistics. Ministry of Health, 2015 (https://www.health.govt.nz/publications/tatau-kahukura-maori-health-chart-book-2015-3rd-edition).

[13] Waitangi Tribunal. *He Whakaputanga me te Tiriti*. Waitangi Tribunal, 2014 (https://forms.justice.govt.nz/search/Documents/WT/wt_DOC_85648980/Te%20RakiW_1.pdf).

[14] Waitangi Tribunal. Hauora: Report on Stage One of the Health Services and Outcomes Kaupapa Inquiry. Waitangi Tribunal, 2023 (https://forms.justice.govt.nz/search/Documents/WT/wt_DOC_195476216/Hauora%202023%20W.pdf).

[15] Haitana T, Pitama S, Cormack D, Clark MTR, Lacey C. ‘It absolutely needs to move out of that structure’: Māori with bipolar disorder identify structural barriers and propose solutions to reform the New Zealand mental health system. Ethn Health 2023; 28(2): 234–56. 35040732 10.1080/13557858.2022.2027884

[16] Graham R, Masters-Awatere B. Experiences of Māori of Aotearoa New Zealand's public health system: a systematic review of two decades of published qualitative research. Aust N Z J Public Health 2020; 44(3): 193–200. 32311187 10.1111/1753-6405.12971

[17] Cunningham R, Stanley J, Haitana T, Pitama S, Crowe M, Mulder R, et al. The physical health of Māori with bipolar disorder. Aust N Z J Psychiatry 2020; 54(11): 1107–14. 32929981 10.1177/0004867420954290

[18] Monk NJ, Cunningham R, Stanley J, Crengle S, Fitzjohn J, Kerdemelidis M, et al. The physical health and mortality of Māori following first episode psychosis diagnosis: a 15-year follow-up study. Aust N Z J Psychiatry [Epub ahead of print] 21 Aug 2024. Available from: 10.1177/00048674241270981. PMC11529131 39169471

[19] Manuel J, Cunningham R, Gibb S, Petrović-van der Deen FS, Porter RJ, Pitama S, et al. Non-indigenous privilege in health, justice and social services preceding first episode psychosis: a population-based cohort study. Aust N Z J Psychiatry 2023; 57(6): 834–43. 36002996 10.1177/00048674221119964

[20] Petrović-van der Deen FS, Cunningham R, Manuel J, Gibb S, Porter RJ, Pitama S, et al. Exploring indigenous ethnic inequities in first episode psychosis in New Zealand – a national cohort study. Schizophr Res 2020; 223: 311–8. 32948382 10.1016/j.schres.2020.09.004

[21] Galletly C, Castle D, Dark F, Humberstone V, Jablensky A, Killackey E, et al. Royal Australian and New Zealand College of Psychiatrists clinical practice guidelines for the management of schizophrenia and related disorders. Aust N Z J Psychiatry 2016; 50(5): 410–72. 27106681 10.1177/0004867416641195

[22] Pringsheim T, Kelly M, Urness D, Teehan M, Ismail Z, Gardner D. Physical health and drug safety in individuals with schizophrenia. Can J Psychiatry 2017; 62(9): 673–83. 28718324 10.1177/0706743717719898 PMC5593246

[23] Ministry of Health. Cardiovascular Disease Risk Assessment and Management for Primary Care. Ministry of Health, 2018 (https://www.tewhatuora.govt.nz/publications/cardiovascular-disease-risk-assessment-and-management-for-primary-care/).

[24] Goddard-Nash A, Makate M, Varhol R, Quirk F, Larsen R, McGeoch G, et al. Evaluation of HealthPathways: an appraisal of usage, experiences and opinions of healthcare professionals in Australia and New Zealand. Aust Health Rev 2020; 44(4): 590–600. 32693906 10.1071/AH19214

[25] NHS England. Improving the Physical Health of People Living with Severe Mental Illness. NHS England, 2024 (https://www.england.nhs.uk/long-read/improving-the-physical-health-of-people-living-with-severe-mental-illness/).

[26] Manuel J, Pitama S, Clark MTR, Crowe M, Crengle S, Cunningham R, et al. Racism, early psychosis and institutional contact: a qualitative study of indigenous experiences. Int Rev Psychiatry 2023; 35(3–4): 323–30. 37267030 10.1080/09540261.2023.2188074

[27] Haitana T, Pitama S, Cormack D, Clark MTR, Lacey C. ‘If we can just dream…’ Māori talk about healthcare for bipolar disorder in New Zealand: a qualitative study privileging Indigenous voices on organisational transformation for health equity. Int J Health Plann Manage 2022; 37(5): 2613–34. 35460284 10.1002/hpm.3486 PMC9546144

[28] Haitana T, Pitama S, Cormack D, Clark MTR, Lacey C. Culturally competent, safe and equitable clinical care for Ma¯ori with bipolar disorder in New Zealand: the expert critique of Ma¯ori patients and Wha¯nau. Aust N Z J Psychiatry 2022; 56(6): 648–56. 34263663 10.1177/00048674211031490 PMC9131406

[29] Te Whatu Ora/Health New Zealand. *PRIMHD - Mental Health Data*. New Zealand Government, 2024 (https://www.tewhatuora.govt.nz/our-health-system/data-and-statistics/nz-health-statistics/national-collections-and-surveys/collections/primhd-mental-health-data/).

[30] Cunningham R, Peterson D, Sims A. Specialist mental health care for older adults in New Zealand-an exploration of service models and routine data. N Z Med J 2019; 132(1489): 30–8. 30703777

[31] Te Whatu Ora/Health New Zealand. *Data Protection and Privacy*. New Zealand Government, 2024 (https://www.tewhatuora.govt.nz/our-health-system/data-and-statistics/nz-health-statistics/data-protection-and-privacy/).

[32] Te Whatu Ora/Health New Zealand. *Collections*. New Zealand Government, 2023 (https://www.tewhatuora.govt.nz/our-health-system/data-and-statistics/nz-health-statistics/national-collections-and-surveys/collections/).

[33] Ministry of Health. HISO 10001:2017 Ethnicity Data Protocols. Ministry of Health, 2017 (https://www.tewhatuora.govt.nz/assets/Our-health-system/Digital-health/Health-information-standards/hiso_10001-2017_ethnicity_data_protocols_21_apr.docx).

[34] Te Whatu Ora/Health New Zealand. *Laboratory Claims Collection*. New Zealand Government, 2024 (https://www.tewhatuora.govt.nz/our-health-system/data-and-statistics/nz-health-statistics/national-collections-and-surveys/collections/laboratory-claims-collection/).

[35] Stanley J, Sarfati D. The new measuring multimorbidity index predicted mortality better than Charlson and Elixhauser indices among the general population. J Clin Epidemiol 2017; 92: 99–110. 28844785 10.1016/j.jclinepi.2017.08.005

[36] Atkinson J, Salmond C, Crampton P. NZDep2018 Index of Deprivation, Interim Research Report, December 2019. University of Otago, 2019 (https://www.otago.ac.nz/__data/assets/pdf_file/0025/327481/nzdep2018-index-of-deprivation-research-report-interim-dec-2019-730394.pdf).

[37] Vandenbroucke JP, Elm E, Altman DG, Gøtzsche PC, Mulrow CD, Pocock SJ, et al. Strengthening the reporting of observational studies in epidemiology (STROBE): explanation and elaboration. Ann Intern Med 2007; 147(8): W163-94. 17938389 10.7326/0003-4819-147-8-200710160-00010-w1

[38] Armitage R. Where Are We with Physical Health Checks for People with Severe Mental Illness in the #Newnormal? *BJGPLife*, 2023 (https://bjgplife.com/where-are-we-with-physical-health-checks-for-people-with-severe-mental-illness-in-the-newnormal/).

[39] Jeffreys M, Ellison-Loschmann L, Irurzun-Lopez M, Cumming J, McKenzie F, on behalf of the Primary Health Care Programme Grant T. Financial barriers to primary health care in Aotearoa New Zealand. Fam Pract [Epub ahead of print] 11 Sep 2023. Available from: 10.1093/fampra/cmad096. PMC11636556 37696758

[40] Te Whatu Ora/Health New Zealand. Quality Standards for Diabetes Care. Te Whatu Ora/Health New Zealand, 2020 (https://www.tewhatuora.govt.nz/for-health-professionals/clinical-guidance/diseases-and-conditions/long-term-conditions/diabetes/quality-standards-for-diabetes-care-2020/#:~:text=All%20people%20with%20diabetes%3A,are%20appropriate%20and%20locally%20available).

[41] Pitama S, Robertson P, Cram F, Gillies M, Huria T, Dallas-Katoa W. Meihana model: a clinical assessment framework. N Z J Psychol 2007; 36(3): 118.

[42] Lacey C, Huria T, Beckert L, Gilles M, Pitama S. The Hui process: a framework to enhance the doctor-patient relationship with Maori. N Z Med J 2011; 124(1347): 72–8. 22237570

[43] Ministry of Health. Cabinet and Briefing Material: Disestablishment of the Māori Health Authority. Ministry of Health, 2024 (https://www.health.govt.nz/about-ministry/information-releases/release-ministerial-decision-making-documents/cabinet-and-briefing-material-disestablishment-maori-health-authority).

[44] Taipale H, Tanskanen A, Mehtälä J, Vattulainen P, Correll CU, Tiihonen J. 20-year follow-up study of physical morbidity and mortality in relationship to antipsychotic treatment in a nationwide cohort of 62,250 patients with schizophrenia (FIN20). World Psychiatry 2020; 19(1): 61–8. 31922669 10.1002/wps.20699 PMC6953552

[45] Solmi M, Tiihonen J, Lähteenvuo M, Tanskanen A, Correll CU, Taipale H. Antipsychotics use is associated with greater adherence to cardiometabolic medications in patients with schizophrenia: results from a nationwide, within-subject design study. Schizophr Bull 2022; 48(1): 166–75. 34286338 10.1093/schbul/sbab087 PMC8781351

[46] Correll CU, Solmi M, Croatto G, Schneider LK, Rohani-Montez SC, Fairley L, et al. Mortality in people with schizophrenia: a systematic review and meta-analysis of relative risk and aggravating or attenuating factors. World Psychiatry 2022; 21(2): 248–71. 35524619 10.1002/wps.20994 PMC9077617

[47] Codyre D, Sharon C, Didsbury L, Henry R, Crozier D. *Te Tumu Waiora: The Integrated Primary Mental Health and Addiction Model. New Zealand Doctor*, 2021 (https://www.nzdoctor.co.nz/sites/default/files/2021-06/f36ea340-f60a-475f-aa5d-ad21ca164752.pdf).

[48] Shiers D, Curtis J. Cardiometabolic health in young people with psychosis. Lancet Psychiatry 2014; 1(7): 492–4. 26361295 10.1016/S2215-0366(14)00072-8

[49] Harris RB, Paine S-J, Atkinson J, Robson B, King PT, Randle J, et al. We still don't count: the under-counting and under-representation of Māori in health and disability sector data. N Z Med J 2022; 135(1567): 54–7. 10.26635/6965.5849 36521086

[50] Lee J. Decolonising Māori narratives: Pūrākau as a method. MAI Rev 2009; 2: 3.

