# Supplementary material for: Inequity in cardiometabolic hospital admissions and blood screening in New Zealand Indigenous Māori with psychosis
Source: BJPsych Open. 2024 Sep 24;10(5):e159. doi: 10.1192/bjo.2024.759 (PMC11457196; doi:10.1192/bjo.2024.759)
Supplement: Monk et al. supplementary material [file S2056472424007592sup001.docx]

**Inequity in cardiometabolic hospital admissions and blood screening in New Zealand Indigenous Māori with psychosis**

Nathan J. Monk, Ruth Cunningham, James Stanley, Julie Fitzjohn, Melissa Kerdemelidis, Helen Lockett, Andre D. McLachlan, Richard J. Porter, Waikaremoana Waitoki, Cameron Lacey

**Supplementary materials**

| Table S1. Diagnostic codes for psychotic disorders | | |
| --- | --- | --- |
| Psychotic disorder | ICD-9/DSM-IV | ICD-10 |
| Schizophrenia | 29510-29569, 29580-29595 | F20 |
| Bipolar I | 29600-29602, 29604-29606, 29640-29642, 29644-29646, 29650-29652, 29654-29656, 29660-29662, 29664-29666, 2967, 29680, 29689 | F302-F309, F312, F313, F315, F316 |
| Schizoaffective | 29570-29575 | F25 |
| Depression with psychosis | 29624, 29634 | F323, F333 |
| Substance with psychosis | 2913-2915, 29211-29212 | F105, F115, F125, F135, F145, F155, F165, F175, F185, F195 |
| Other | 2988, 2971, 2973 | F22-F24, F28 |
| Organic | 29381, 29382 | F060, F062 |
| Unspecified | 2989 | F29 |
|  | | |

| Table S2. Diagnostic codes for cardiovascular disease (CVD) | | |
| --- | --- | --- |
| CVD condition | ICD-9 | ICD-10 |
| Myocardial infarction | 410 | I210-I214, I219-I221, I228, I229 |
| Unstable angina | - | I200 |
| Other coronary heart disease | 41189, 4148, 4275, 4296, 4139, 4131, 4295, 4148, 41410, 41419, 412, 42971, 42979, 41411, 41412, 41181 | I236, I248, I249, I255, I46, I235, I209, I201, I234, I256, I253, I252, I232, I230, I254, I233, I208, I240, I238, I231 |
| Ischaemic stroke | 433, 434, 436 | I63, I64 |
| Haemorrhagic stroke | 430, 431, 432 | I60, I61 |
| Transient ischaemic attack | 4350, 4351, 4352, 4353, 4358, 4359, 4378, 36234 | G450, G451, G452, G453, G458, G459, G46 |
| Peripheral vascular disease | 25070, 25071, 25072, 25073, 44381, 4410, 4411, 4413, 4415, 4416, 4439, 444 | E1050, E1051, E1052, E1150, E1151, E1152, E1451, E1452, I7021, I7022, I7023, I7024, I710, I711, I713, I715, I718, I739, I74 |
| Congestive heart failure | 40201, 40211, 40291, 40401, 40411, 40491, 40403, 40413, 40493, 428 | I110, I130, I132, I50 |
| Other ischaemic CVD-related codes | 41400, 41401, 4141, 4142, 4143, 4144, 4148, 4149, 4275, 44329, 4370, 438, 4400, 4401, 4402, 4404, 4408, 4409, 441, V4581, V4582, V4502, V4321, V4322, V4509, V4500 | E1059, E1159, E1459, I250, I251, I258, I259, I46, I65, I66, I670, I672, I690, I691, I693, I694, I698, I700, I701, I702, I708, I709, I714, Z951, Z955, Z958, Z959 |

| Table S3. Diagnostic codes for comorbid non-psychotic psychiatric disorders | | |
| --- | --- | --- |
| Psychiatric disorder | ICD-9/DSM-IV | ICD-10 |
| Depression | 311 | F320, F321, F322, F324, F325, F328, F329, F32A, F330, F331, F332, F3340, F334, F338, F339 |
| Anxiety | 30000-30009, 30020-30029 | F40, F41 |
| Substance | 2910-2912, 2919, 29181, 29182, 2920, 2922, 2929, 29281-29289 | F100-F199 (excluding all codes ending in “5”, e.g. F105, F115, etc) |
| Personality | 3010, 30110-30113, 3013, 3014, 30150-30159, 3016, 3017, 30181-30189, 3019 | F60, F61, F62 |
|  | | |

| Table S4. Risks of hospitalisation, mortality, and blood screening events for Māori (*n* = 7274), Pacific Peoples (*n* = 1769), and NZ European (*n* = 9702) during study period | | | | | | | |
| --- | --- | --- | --- | --- | --- | --- | --- |
|  | Māori *n* (%) | Pacific *n* (%) | NZ European *n* (%) | Māori Crude OR (95% CI)^a^ | Māori Adjusted OR (95% CI)^b^ | Pacific Crude OR (95% CI) | Pacific Adjusted OR (95% CI) |
| Physical health admission | 1786 (24.6) | 441 (24.9) | 2566 (26.5) | 0.91 (0.84, 0.97) | 1.06 (0.98, 1.14) | 0.92 (0.82, 1.04) | 1.04 (0.92, 1.17) |
| External cause admission | 1570 (21.6) | 387 (21.9) | 2309 (23.8) | 0.88 (0.82, 0.95) | 0.97 (0.90, 1.05) | 0.90 (0.79, 1.01) | 0.96 (0.85, 1.09) |
| Cardiovascular admission | 168 (2.3) | 47(2.7) | 194 (2.0) | 1.16 (0.94, 1.43) | 1.78 (1.42, 2.22) | 1.34 (0.96, 1.83) | 1.86 (1.31, 2.60) |
| Diabetes admission | 427 (5.9) | 139 (7.9) | 376 (3.9) | 1.55 (1.34, 1.78) | 2.13 (1.83, 2.49) | 2.12 (1.72, 2.58) | 2.79 (2.24, 3.46) |
| Mortality | 163 (2.2) | 41 (2.3) | 229 (2.4) | 0.95 (0.77, 1.16) | 1.21 (0.97, 1.49) | 0.98 (0.69, 1.36) | 1.23 (0.86, 1.72) |
| Lipids screening | 4373 (60.1) | 1274 (72.0) | 5845 (60.2) | 0.99 (0.93, 1.06) | 1.23 (1.15, 1.32) | 1.70 (1.52, 1.90) | 2.04 (1.81, 2.30) |
| HbA1c screening | 4770 (65.6) | 1325 (74.9) | 6538 (67.9) | 0.92 (0.86, 0.98) | 1.13 (1.06, 1.22) | 1.44 (1.29, 1.62) | 1.75 (1.55, 1.97) |
| Notes: ^a^ NZ European are the reference group for all odds ratios; ^b^ Models adjusted for age, gender, and socioeconomic deprivation | | | | | | | |
